# Immature Plasma Cell Myeloma Mimics Metastatic Renal Cell Carcinoma on ^18^F-PSMA-1007 PET/CT Due to Endothelial PSMA-Expression

**DOI:** 10.3390/diagnostics11030423

**Published:** 2021-03-03

**Authors:** Lena M. Mittlmeier, Stephan T. Ledderose, Melanie Schott, Matthias Brendel, Leonie Beyer, Sebastian Theurich, Doris Mayr, Christoph Walz, Wolfgang G. Kunz, Jens Ricke, Peter Bartenstein, Harun Ilhan, Michael Staehler, Marcus Unterrainer

**Affiliations:** 1 Department of Nuclear Medicine, University Hospital, LMU Munich, 81377 Munich, Germany; Lena.mittlmeier@med.uni-muenchen.de (L.M.M.); Matthias.Brendel@med.uni-muenchen.de (M.B.); Leonie.Beyer@med.uni-muenchen.de (L.B.); Peter.Bartenstein@med.uni-muenchen.de (P.B.); Harun.Ilhan@med.uni-muenchen.de (H.I.); 2 Institute of Pathology, LMU Munich, 81377 Munich, Germany; Stephan.Ledderose@med.uni-muenchen.de (S.T.L.); Doris.Mayr@med.uni-muenchen.de (D.M.); Christoph.Walz@med.uni-muenchen.de (C.W.); 3 Department of Urology, University Hospital, LMU Munich, 81377 Munich, Germany; Melanie.Schott@med.uni-muenchen.de (M.S.); Michael.Staehler@med.uni-muenchen.de (M.S.); 4 Department of Medicine III, University Hospital, LMU Munich, 81377 Munich, Germany; Sebastian.theurich@med.uni-muenchen.de; 5 Department of Radiology, University Hospital, LMU Munich, 81377 Munich, Germany; Wolfgang.Kunz@med.uni-muenchen.de (W.G.K.); Jens.Ricke@med.uni-muenchen.de (J.R.)

**Keywords:** renal cell carcinoma, ^18^F-PSMA PET/CT, myeloma

## Abstract

We present a 71-year-old female patient who underwent ^18^F-PSMA-1007 PET/CT for suspected metastatic renal cell carcinoma (RCC), as RCC also shows high PSMA-expression in tumor neovascularization. ^18^F-PSMA-1007 PET/CT showed a high PSMA-avidity in the renal tumor, enlarged intra-abdominal and mediastinal lymph nodes. Moreover, PSMA-positive pleural, pulmonal and osseous lesions were found. However, histopathology revealed an immature plasma cell myeloma with an endothelial PSMA-expression of the neovasculature. This case illustrates the increased PSMA-avidity in multiple myeloma and highlights PSMA as a potential theragnostic target in multiple myeloma. For clinical routine, lymphatic diseases such as extramedullary myeloma should be considered as differential diagnosis in PSMA-avid renal masses on PET/CT.

A 71-year-old woman presented to the uro-oncological department with a newly diagnosed, left-sided renal tumor for further clinical workup and the subsequent initiation of therapy. Contrast-enhanced computed tomography (CT), initially performed at the pelvis and the lower limbs to rule out arterial occlusion, revealed an incidental finding of an extensive, inhomogeneous, marginal contrast-enhancing renal tumor of the left kidney. In addition, there were surrounding, pathologically enlarged abdominal lymph nodes (Figure 1A). The primary differential diagnosis consisted of renal cell carcinoma (RCC) with nodal abdominal spread. As the initial results highlighted the added clinical value of prostate-specific-antigen (PSMA)-targeted positron emission tomography (PET) imaging in metastatic RCC due to the PSMA-expression of tumor neovascularization [1,2,3,4,5], this patient underwent ^18^F-PSMA-1007 PET/CT for whole body staging before any further tumor-specific therapies. Here, the left renal lesion showed a markedly increased PSMA-expression (maximal standardized uptake value (SUV_max_) 30.0; tumor-to-background ratio (TBR), SUV_max_/SUV_mean-liver_ 3,1; Figure 1B).

The pre-known enlarged lymph nodes also showed a high PSMA-avidity on PET (SUV_max_ 17.0). In addition, mediastinal lymph nodes, multilocular pulmonary, pleural, and osseous lesions presented with PSMA-avidity, partially even with extra-osseous soft tissue extension and mixed sclerotic/lytic correlate on CT. Beyond this, a left para-ovarian soft tissue bulk with highly increased PSMA-expression was found (Figure 1C,D). A MIP projection demonstrates the whole tumor burden (Figure 1E). For further evaluation, histological specimens of the left renal tumor were obtained using ultrasound-guided biopsy.

The microscopic examination of the biopsy revealed dense aggregates of mature and immature plasma cells in hematoxylin and eosin (H&E)-stained tissue specimens (Figure 1F) with positivity for CD38 and CD138 and strong nuclear staining for multiple myeloma oncogene 1 (MUM1) (Figure 1G). Staining for pan-cytokeratin (KL1 staining) was negative, ruling out a plasmacytoid urothelial carcinoma of the renal pelvis (Figure 1H). The lesion was negative for CD20, cytokeratin-7 (CK7), paired box gene 8 (Pax8), and GATA3. Limitations in biopsy quantity and quality prevented the immuno-histochemical analysis of kappa and lambda light chains; however, based on histomorphology and the typical expression profile the diagnosis was confirmed as an immature plasma cell myeloma independently by two experienced pathologists. Immunohistochemical staining for PSMA showed a strong expression in tumor-associated microvasculature (Figure 1I).

To date, increased PSMA-avidity in multiple myeloma has been described only sporadically in the literature [6,7,8] Of note, in a single case, PSMA expression was additionally shown to decrease after systemic therapy, highlighting PSMA-expression as a potential theranostic target for multiple myeloma, e.g., during radioligand therapy using ^177^Lu- or ^225^Ac-labeled PSMA-ligands [9]. To date, however, there is no literature describing PSMA-avid masses suggestive of renal RCC, which was then confirmed to be highly PSMA-avid multiple myeloma. For clinical routine, this case underlines that lymphatic diseases such as extramedullary myeloma should be considered as differential diagnosis in PSMA-avid renal masses on PET/CT despite their rare occurrence, as manifestations of multiple myeloma may also show a highly endothelial PSMA-expression.

## Figures and Tables

**Figure 1:**
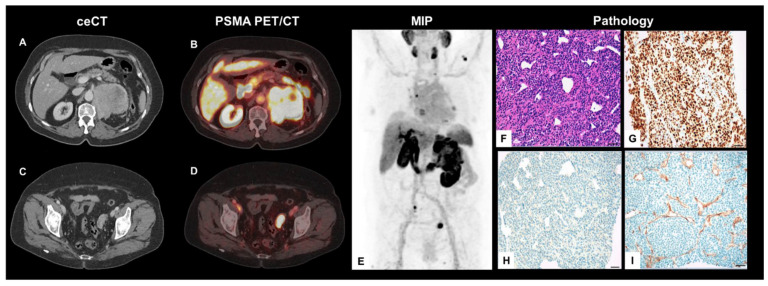
Axial, contrast-enhanced CT (ceCT) (**A**,**C**) and fused PET/CT planes (**B**,**D**), MIP (**E**) as well as histology findings from ultrasound-guided biopsy (**F**–**I**) (Scale bar = 50 µm).

## Data Availability

The data presented in this study are available on reasonable request from the corresponding author.
